# Association of Body Mass Index with the Tuberculosis Infection: a Population-based Study among 17796 Adults in Rural China

**DOI:** 10.1038/srep41933

**Published:** 2017-02-08

**Authors:** Haoran Zhang, Xiangwei Li, Henan Xin, Hengjing Li, Mufei Li, Wei Lu, Liqiong Bai, Xinhua Wang, Jianmin Liu, Qi Jin, Lei Gao

**Affiliations:** 1 MOH Key Laboratory of Systems Biology of Pathogens, Institute of Pathogen Biology, and Centre for Tuberculosis, Chinese Academy of Medical Sciences and Peking Union Medical College, Beijing, 100730, China; 2 Jiangsu Provincial Center for Diseases Control and Prevention, Nanjing, 210009, China; 3 Hunan Provincial Institute of Tuberculosis Prevention and Control, Changsha, 410006, China; 4 Gansu Provincial Center for Diseases Control and Prevention, Lanzhou, 730000, China; 5 The Sixth People’s Hospital of Zhengzhou, Zhengzhou, 450061, China

## Abstract

Body mass index (BMI) has been shown to be associated with host susceptibility to several infections. However, the link between BMI and the risk of tuberculosis (TB) infection has been sparsely studied in China and in worldwide. Based on the baseline survey of a population-based, prospective study in rural China, the association between BMI and TB infection among adults was estimated by means of cross-sectional analysis. TB infection status was tested using QuantiFERON-TB Gold In-Tube (QFT), a commercial of interferon-γ release assay (IGRA). Totally, 17796 eligible participants aged ≥18 years from 4 study sites, were included in the analysis. 21.76% (3873/17796) were observed to be QFT positive. Age and gender standardized prevalence ranged from 16.49% to 23.81% across the study sites. 42.19% study participants were obese/overweight with BMI ≥ 24.0 kg/m^2^. BMI ≥ 28.0 kg/m^2^ was observed to be independently associated with QFT positivity (adjusted odds ratio: 1.17, 95% confidence interval: 1.04–1.33). The strength of the association was found to be geographically diversity, which might be explained, at least partly, by the varied local TB epidemic status. Our results suggest that individuals with obesity might be one important target population for TB infection control in rural China.

Latent tuberculosis infection (LTBI) screening and preventive treatment among high risk populations has been a major component of tuberculosis (TB) control programs in low-burden countries, such as USA and Canada^1^. For the areas and countries in developing LTBI management guidelines, including China, identification of individuals susceptible to infection and those with highest likelihood of progression to active disease should be the first important issue for consideration. According to testing of TB infection, there are two available methods, Tuberculin Skin Test (TST) and Interferon-γ Gamma Release Assays (IGRAs). The TST measures type IV hypersensitivity in response to purified protein derivative (PPD), while IGRAs detect interferon–γ (IFN-γ) level after the *in-vitro* stimulation with specific *Mycobacterium tuberculosis* (MTB) antigens^2^. However, the longitudinal data provided compelling evidence that the TST results were influenced by several factors including Bacillus Calmette-Guerin (BCG) vaccination and old age^3^. According to high-risk populations for screening and prophylactic treatment of LTBI, only close contacts of patients with active pulmonary TB and HIV infections are recommended currently in China^4^.

Globally, risk factors of TB infection prevalence have been found to be varied across areas. Low body mass index (BMI) has been shown to be associated with host susceptibility to active TB development^5^,^6^. In addition, obesity (BMI ≥ 30 kg/m^2^) and overweight (25 kg/m^2^ ≤ BMI < 30 kg/m^2^) were observed to be significantly associated with decreased risk of developing active TB as compared with normal-weight (18.5 kg/m^2^ ≤ BMI < 25 kg/m^2^)^7^. Additionally, type 2 diabetes mellitus (T2DM) has been suggested to be a re-emerging risk factor for TB development^8^ and for TB infection as well^9^. However, the link between BMI and the risk for TB infection has not been widely studied in worldwide. Therefore, the present study aims to assess the association of BMI with TB infection in rural adults based on the baseline data of a population-based multi-center prospective study from China.

## Results

### Characteristics of the study participants

**Figure 1:**
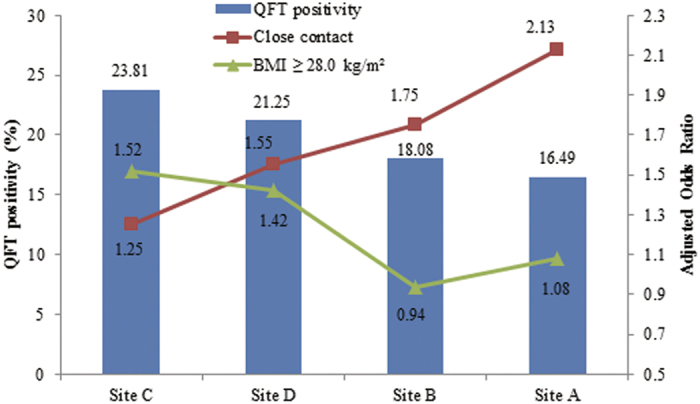
Age and gender-standardized prevalence of QFT positivity by study site. The associations of QFT positivity with close contact with active TB patients and BMI ≥ 28.0 kg/m^2^ were shown by study site as well. The strength of the association for close contact was strongest at the site with the lowest prevalence of infection. Inversely, the association of BMI ≥ 28.0 kg/m^2^ was strongest for the site with the highest prevalence of infection.

A total of 17796 eligible participants were included in this analysis. Basic characteristics of the study population were shown in Table 1. More than a half (55.03%) of them were females and three quarters (74.29%) were older than 40 years. Gender and age distributions differed significantly across study sites (p < 0.0001). As compared to the participants from the other three study sites, participants from Site C showed lower educational levels (89.21% with middle school levels or lower) and higher exposure to close contact with TB patients (12.83%). Nearly one third of the participants reported ever smoking (29.16%) and about one fifth reported alcohol drinking (22.25%). Nearly half of the participants (48.42%) presented a BCG scar. 5.03% participants had a self-reported history of T2DM or with a baseline fast blood glucose level ≥7.0 mmol/L. The median BMI of the participants was 23.19 kg/m^2^ (interquartile range [IQR]: 21.06–25.76 kg/m^2^) and 42.19% study participants were overweight or obese with BMI ≥ 24.0 kg/m^2^. The distributions of BMI across age groups and study sites by gender please refer to Supplementary Tables S1 and S2. Among the study population, 21.76% were QuantiFERON-TB Gold In-Tube (QFT; a commercial IGRA kit) positive. After standardization for age and gender, the QFT positivity prevalence was found to be varied from 16.49% for Site A to 23.81% for Site C (Fig. 1).

### Factors independently associated with QFT positivity

For the association analysis (Table 2), QFT positivity has been observed to be associated with study site, age, gender, education level, smoking, close contact with TB patients, low density lipoprotein (LDL) level and BMI. History of T2DM was not observed to be related with QFT positivity. In addition, as shown in Table 3, BMI ≥ 28.0 kg/m^2^ (obesity) was observed to be independently associated with QFT positivity (with an adjusted odds ratio [OR]: 1.17, 95% confidence interval [CI]: 1.04–1.33). Male sex, increasing age, ever smoked, close contact with TB patients, and LDL level were also independently related with QFT positivity.

### Association between QFT positivity and BMI (kg/m^2^) categories by sites

The association between BMI and QFT positivity was found to be geographically diverse as shown in Table 4. The strength of the association between QFT positivity and BMI ≥ 28.0 kg/m^2^ were statistically significant for Site C (adjusted OR: 1.52, 95% CI: 1.20–1.94) and Site D (adjusted OR: 1.42, 95% CI: 1.05–1.91).

### Stratified analysis of the covariates on the association of BMI (kg/m^2^) with QFT positivity

In Table 5, potential impact of other independent factors on the relation of BMI with QFT positivity was evaluated by means of stratified analysis. Participants who were females (adjusted OR: 1.26, 95% CI: 1.08–1.48) or older than 60 years (adjusted OR: 1.31, 95% CI: 1.07–1.60) were found to be under much higher risk of QFT positivity if they were BMI ≥ 28.0 kg/m^2^.

## Discussion

In this population-based, multicenter study conducted in rural China, BMI ≥ 28.0 kg/m^2^ was found to be independently associated with host susceptibility to TB infection. The strength of such an association was stronger in the study sites with higher prevalence of TB infection. The increased risk of TB infection among individuals with obesity was observed to be modified by other potential independent factors such as gender, age and smoking. High risk populations for TB infection, such as elderly with obesity found in this study, should be paid more attention as potential target populations for TB infection monitoring and preventive intervention in China.

The association between BMI and TB infection has not been comprehensively understood, despite years of research on its links with active TB disease^10^,^11^. Our study provided an opportunity, with large sample size and different study sites with various TB epidemics, to explore the relation between BMI and TB infection in the general rural population in China. In addition, IGRA has been approved by the American Centers for Disease Control and Prevention (CDC) as an alternative screening strategy to TST for LTBI testing currently^12^. Based on our previous results, the TST results were found to be influenced by several factors including old age and the status of BCG vaccination^13^. Therefore, the present study used IGRAs rather than TST to define the status of TB infection. Participants aged 18 years or lower were excluded in the present study to minimize the potential bias caused by the dynamic change in BMI for growing children.

Contrary to the link between lower BMI and increased risk of active TB^14^, our results showed that BMI ≥ 28.0 kg/m^2^ was independently associated with TB infection. It has been suggested that excess adiposity negatively impacts immune function and host defense in obese individuals^15^,^16^,^17^. Additionally, the accumulation of adipose tissue might attenuate host pulmonary defense through metabolic disturbances^18^,^19^. Therefore, the potential mechanism underlying the relation of obesity and TB infection might be that adipose tissue takes effect through a variety of immune mediators^5^. Animal results proved that leptin-deficient (*ob/ob*) mice were highly susceptible to pulmonary TB infection^20^. The prevalence of TB infection prevalence increased gradually from 18.50% for underweight subjects to 23.67% for obese subjects in the present study would be consistent with the above speculation. If BMI is causally linked to the risk of latent TB in the way that our data presented here suggest, then promoting weight loss in overweight or obese populations and shifting the overall BMI distribution to lower values, would further reduce latent TB incidence, especially for people who may become TB infected. However, due to the limitation of cross-sectional study design, it should be critical to confirm the directionality of association between BMI and TB infection. In addition, we could not exclude another possibility that individuals with low BMI were under higher risk of developing active disease and then those with normal and high BMI stayed latent infection. Therefore, prospective studies were needed to clarify the underlying mechanisms for the observed relation between obesity and increased risk of TB infection. To put this in context, our data might strengthen the rationale to assess and implement strategies aimed at stopping increasing overweight and obesity trends in China and mitigating their public health effects.

In the present study, the strength of the association between BMI and TB infection was found to be geographically diverse. As shown in Fig. 1, the prevalence of TB infection in Site A was much lower than the other three sites, TB infection showed stronger association with close contact with active TB patients but relatively modest relation to BMI. It suggests that the effect of BMI on TB infection might be neglected among regions with lower TB epidemics. At the same time, certain regional disparities in TB prevalence suggest that screening and identification of LTBI in contacts of TB patients might be seen as a good clinical practice in low-endemic settings and tuberculosis-control activities should be regularly evaluated for effectiveness against changing epidemiological information. In addition, such geographical diversity might be explained by different distributions of the other covariates associated with TB infection such as age, gender and smoking.

Given that obesity is one main cause of T2DM, it has given strength about the pertinence of this study. Data showed that 201 (23.16%) of 868 participants with a history of T2DM had the status of BMI ≥ 28.0 kg/m^2^ (Supplementary Table S3). However, logistic regression analysis revealed that the relation between T2DM and TB infection was non-significant in our study population. It might further suggest that high BMI was independently correlated with TB infection. Although T2DM is a cause of comorbidity for people with active TB^21^,^22^, its role in TB infection need further explored.

Due to the fact that our data were cross-sectional design based, our study has several limitations. First, the causal nature between BMI and QFT positivity is uncertain, even the biologic mechanism is more plausible to support attenuated immune function and increased susceptibility to infections for individuals with obesity. Second, some covariant factors such as history of close contact with TB patients were collected by a face-to-face interviewed questionnaire. Potential recall bias caused by inaccurate response could therefore not be excluded. Silicosis and HIV infection have been suggested to be potential risk factors for TB infection^9^, however, no participant in our study was self-reported or officially registered patients with silicosis and/or HIV infection. Low prevalence of silicosis and HIV infection in the general rural population in China^23^,^24^,^25^ limited our analysis to explore their potential impact on TB infection. Third, a standard 0.35 IU/mL cutoff was used for QFT in this study which might not be the most appropriate cutoff for this population, because it has not been extensively validated in China. Finally, suspected cases with active TB based on the diagnosis of chest X-ray were excluded from the prevalence analysis of TB infection in the present study. Considering lacking pathogenic evidence, such as sputum smear, sputum culture or gene X-pert test, potential bias caused by misclassification of disease status could not be completely excluded. Despite these limitations, our results provide valuable insight into the relation of BMI with infectious diseases.

## Conclusions

In summary, a positive association between obesity and TB infection was observed in our study population. It suggests that BMI management should be attached attention for the public health including infectious diseases control in China. In a community level, high-risk subgroups including the elderly with obesity found in this study should be prioritized for TB infection control in China and areas with a similar epidemiological profile.

## Methods

### Ethics consideration

Ethics approval was obtained from the ethics committee of the Institute of Pathogen Biology, Chinese Academy of Medical Sciences (Beijing, China) (No: IPB-2013–5). All experiments and study procedures were performed in accordance with relevant guidelines and regulations, and including any relevant details. All participants gave written informed consent.

### Study design and participants

A population-based, prospective cohort study (LATENTTB-NSTM) addressing TB infection and active disease development was conducted at four study sites (Site A: eastern China, plains; Site B: central China, plains; Site C: western China, hills; or Site D: western China, basin.) in rural China during 2013–2015. Registered rural residents (5 years older) at the four study sites were the target population of the study. The baseline survey was conducted during July to September 2013, which has been reported in detail previously^13^.

The present study was restricted to the adults participated in the baseline survey. The inclusion criteria were: aged 18 years or older (referenced as June 1, 2013); registered resident or with continuous residence at the study site for ≥6 months over the past year; able to complete the investigations and tests during the study duration; and provision of voluntary written informed consent. The exclusion criteria were: current active TB, self-reported history of TB, and pregnancy.

### Data collection

For each study participant, socio-demographic information was collected by a standardized questionnaire administered by trained interviewers. Data collected included age, gender, educational level, history of close contact with a TB patient, smoking status (never smoked or ever smoked), alcohol use status (never used or ever used) and history of T2DM (self-reported and/or fast blood glucose higher than 7.0 mmol/L at baseline examination)^26^. Ever smoked was defined as those who had smoked more than 5 cigarettes/month. Ever alcohol use was defined as those who had consumed alcohol for the last year. Digital chest radiography (CXR) was performed on all study participants over 15 years of age. Individuals with suspected TB (defined by radiographic abnormalities consistent with active TB) were not included in the LTBI analysis. Height, weight, pulse and the presence of a BCG scar were examined as well.

Venous blood was collected for QFT and blood biochemical examinations, including fast blood glucose, cholesterol, triglyceride, LDL, and high density lipoprotein (HDL) levels. The cutoff value of the blood test index is suggested according to the instruction manual. As a product of IGRA, QFT detected IFN-γ *in vitro* responses to peptide antigens that were associated with MTB infection^27^. Briefly, each QFT consisted of three tubes: (1) a TB antigen tube containing the specific MTB antigens, (2) a mitogen control tube containing a non-specific T-cell-stimulating antigen and serving as a positive control, and (3) a Nil control tube containing no antigens and serving as a negative control. QFT was performed as recommended by the manufacturer using a cutoff value of ≥0.35 IU/ml. The positivity of QFT was defined as TB antigen minus Nil ≥ 0.35 IU/ml and ≥ 25% of Nil value, together with Nil ≤ 8.0 IU/ml according to the manufacturer. The positive results might imply the presence of TB infection.

### Statistical analysis

Questionnaire data, physical examination data (height, weight, pulse, and presence of BCG scar) and laboratory results (QFT and blood biochemical examination) were double entered into a spreadsheet and checked by web-based project-specific data collection and management software. After cleaning, the data were then converted and analyzed using Statistical Analysis System (SAS 9.2; SAS Institute Inc., NC, USA).

BMI was calculated as weight over height squared (kg/m^2^), and was presented as median and IQR when was recognized as continuous variable. BMI was further categorized as underweight (<18.5 kg/m^2^), normal weight (18.5 to 24.0 kg/m^2^), overweight (24.0 to 28.0 kg/m^2^), or obese (≥28.0 kg/m^2^)^28^. It has demonstrated that obesity is in correlation with blood glucose, cholesterol, blood glucose, triglycerides and LDL/HDL^29^,^30^. Therefore, these blood biochemical parameters were included in the analysis. The frequency of categorical variables in the study participants was compared between the study sites using Pearson’s chi-square test. To identify potential variables related with QFT positivity, univariate analysis were performed using Pearson’s chi-square test. All variables with p-values < 0.05 in univariate analysis were entered into the multiple logistic regression analyses, and the associations were then assessed by means of OR and 95% CI. Stepwise backward multiple logistic regression analysis was then used to identify the variables independently associated with QFT positivity. The significance level for the variables stayed in the model was 0.05. To explore the potential modifying effect of the covariates on the association of BMI with QFT positivity, stratified analysis were conducted (BMI was dichotomized at cutoff of 28.0 kg/m^2^).

## Additional Information

**How to cite this article:** Zhang, H. *et al*. Association of Body Mass Index with the Tuberculosis Infection: a Population-based Study among 17796 Adults in Rural China. *Sci. Rep.* **7**, 41933; doi: 10.1038/srep41933 (2017).

**Publisher's note:** Springer Nature remains neutral with regard to jurisdictional claims in published maps and institutional affiliations.

## Supplementary Material

Supplementary Table S1–Table S3

## Figures and Tables

**Table 1:** Characteristics of the study population.

	Total* (N = 17796)	Site A (n = 5068)	Site B (n = 4223)	Site C (n = 4395)	Site D (n = 4110)	p for χ^2^ test
Gender						<0.0001
Male	8003(44.97)	2328(45.94)	2089(49.47)	1991(45.30)	1595(38.81)	
Female	9793(55.03)	2740(54.06)	2134(50.53)	2404(54.70)	2515(61.19)	
Age (years)						<0.0001
18–29	2391(13.44)	472(9.31)	862(20.41)	380(8.65)	677(16.47)	
30–39	2183(12.27)	456(9.00)	566(13.40)	443(10.08)	718(17.47)	
40–49	4651(26.14)	1260(24.86)	1161(27.49)	1084(24.66)	1146(27.88)	
50–59	3613(20.30)	1174(23.16)	746(17.67)	1009(22.96)	684(16.64)	
60–69	3136(17.62)	1069(21.09)	539(12.76)	944(21.48)	584(14.21)	
≥70	1822(10.24)	637(12.57)	349(8.26)	535(12.17)	301(7.32)	
Education level						<0.0001
Primary school or lower	8900(50.01)	2232(44.04)	2001(47.38)	2394(54.47)	2273(55.30)	
Middle school	6197(34.82)	2043(40.31)	1547(36.63)	1527(34.74)	1080(26.28)	
High school	2052(11.53)	608(12.00)	489(11.58)	388(8.83)	567(13.80)	
College or higher	647(3.64)	185(3.65)	186(4.40)	86(1.96)	190(4.62)	
Smoking status						<0.0001
Never smoked	12605(70.84)	3754(74.07)	2878(68.18)	2857(65.01)	3116(75.82)	
Ever smoked	5189(29.16)	1314(25.93)	1343(31.82)	1538(34.99)	994(24.18)	
Alcohol drinking						<0.0001
No	13836(77.75)	3873(76.42)	2851(67.53)	3589(81.66)	3523(85.72)	
Yes	3959(22.25)	1195(23.58)	1371(32.47)	806(18.34)	587(14.28)	
History of close contact with TB patients						<0.0001
No	15826(95.72)	4946(99.32)	3976(96.76)	3473(87.17)	3431(99.13)	
Yes	708(4.28)	34(0.68)	133(3.24)	511(12.83)	30(0.87)	
History of T2DM						<0.0001
No	16396(94.97)	4719(94.12)	3966(95.77)	3758(96.46)	3953(93.83)	
Yes	868(5.03)	295(5.88)	175(4.23)	138(3.54)	260(6.17)	
Number of BCG scars						<0.0001
0	9178(51.58)	3457(68.23)	824(19.51)	3960(96.35)	937(21.32)	
1	5182(29.12)	1254(24.75)	1490(35.28)	143(3.48)	2295(52.23)	
≥2	3434(19.30)	356(7.03)	1909(45.20)	7(0.17)	1162(26.45)	
Cholesterol level^a^						<0.0001
Normal	15065(84.67)	4566(90.13)	3299(78.12)	3122(71.07)	4078(99.22)	
Higher than normal	2727(15.33)	500(9.87)	924(21.88)	1271(28.93)	32(0.78)	
Triglyceride level^b^						<0.0001
Normal	11431(64.25)	3205(63.26)	2376(56.26)	2629(59.85)	3221(78.37)	
Higher than normal	6361(35.75)	1861(36.74)	1847(43.74)	1764(40.15)	889(21.63)	
LDL level^c^						<0.0001
Normal	14942(83.99)	4934(97.41)	3665(86.79)	2708(61.64)	3635(88.44)	
Higher than normal	2849(16.01)	131(2.59)	558(13.21)	1685(38.36)	475(11.56)	
HDL level^d^						<0.0001
Normal	15702(88.25)	4350(85.87)	4193(99.29)	3604(82.04)	3555(86.50)	
Higher than normal	2090(11.75)	716(14.13)	30(0.71)	789(17.96)	555(13.50)	
BMI (kg/m^2^)						
Median (IQR)^‡^	23.29(21.06, 25.76)	23.30(21.25, 25.41)	24.31(21.92, 27.25)	22.93(20.71, 25.54)	22.68(20.58, 25.10)	<0.0001
<18.5	927(5.21)	220(4.34)	127(3.01)	288(6.55)	292(7.10)	<0.0001
18.5–24.0	9362(52.61)	2787(54.99)	1851(43.83)	2384(54.26)	2340(56.93)	
24.0–28.0	5496(30.89)	1629(32.14)	1402(33.20)	1285(29.24)	1180(28.71)	
≥28.0	2010(11.30)	432(8.52)	843(19.96)	437(9.95)	298(7.25)	
QFT test						<0.0001
Negative	13391(75.25)	3962(78.18)	3340(79.09)	3040(69.17)	3049(74.18)	
Positive	3873(21.76)	1052(20.76)	801(18.97)	1173(26.69)	847(20.61)	
Indeterminate	532(2.99)	54(1.07)	82(1.94)	182(4.14)	214(5.21)	

Abbreviations: BMI = body mass index. BCG = Bacillus Calmette-Guerin. HDL = high density lipoprotein. IQR = interquartile range. LDL = low density lipoprotein. QFT = QuantiFERON-TB Gold In-Tube. TB = tuberculosis. T2DM = type 2 diabetes mellitus.

^a,b,c,d^Ranges of normal value for blood tests in different study sites: Cholesterol: Site A = 2.59–5.17 mmol/L, Site B = 3.1–5.17 mmol/L, Site C = 0–5.17 mmol/L, Site D = 0–5.17 mmol/L. Triglyceride: Site A = 0–1.7 mmol/L, Site B = 0.56–1.7 mmol/L, Site C = 0–1.71 mmol/L, Site D = 0–2.3 mmol/L. HDL: Site A = >0.9 mmol/L, Site B = 0.9–2.28 mmol/L, Site C = 1.16–1.55 mmol/L, Site D = 1.05–1.74 mmol/L. LDL: Site A = 0–4.11 mmol/L, Site B = 0-3.36 mmol/L, Site C = 0–3.12 mmol/L, Site D = <3.1 mmol/L.

*Sum might not always be in total because of missing data. Frequency of missing data did not differ significantly between sites. ^‡^Kruskal-Wallis test.

**Table 2:** Univariate and multivariate analysis for QFT positivity.

	n/N (%)	p for χ^2^ test	Adjusted OR* (95% CI)
Study sites		<0.0001	
Site A	1052/5014(20.98)		Reference
Site B	801/4141(19.34)		1.03(0.90–1.17)
Site C	1173/4213(27.84)		1.34(1.19–1.51)
Site D	847/3896(21.74)		1.37(1.21–1.56)
Gender		<0.0001	
Female	1793/9415(19.04)		Reference
Male	2080/7849(26.50)		1.29(1.15–1.44)
Age (years)		<0.0001	
18–29	194/2344(8.28)		Reference
30–39	313/2116(14.79)		1.81(1.47–2.22)
40–49	876/4513(19.41)		2.46(2.04–2.96)
50–59	934/3479(26.85)		3.81(3.16–4.58)
60–69	966/3047(31.70)		4.97(4.10–6.04)
≥70	590/1765(33.43)		5.67(4.61–6.98)
Education level		<0.0001	
Primary school or lower	2175/8573(25.37)		Reference
Middle school	1264/6044(20.91)		1.10(1.00–1.21)
High school	367/2014(18.22)		1.06(0.92–1.22)
College or higher	67/633(10.58)		0.95(0.71–1.26)
Smoking status		<0.0001	
Never smoked	2405/12176(19.75)		Reference
Ever smoked	1468/5086(28.86)		1.28(1.15–1.43)
Alcohol drinking		<0.0001	
No	2877/13373(21.51)		Reference
Yes	996/3890(25.60)		0.99(0.89–1.10)
History of close contact with TB patients		<0.0001	
No	3480/15826(21.99)		Reference
Yes	221/708(31.21)		1.38(1.16–1.64)
History of T2DM			
No	3627/16396(22.12)	Reference	
Yes	246/868(28.34)		1.04(0.88–1.22)
Number of BCG scars			
0	2021/8891(22.73)		Reference
1	1049/5030(20.85)		0.98(0.88–1.10)
≥2	803/3341(24.03)		1.05(0.93–1.20)
Cholesterol level^a^		<0.0001	
Normal	3168/14632(21.65)		Reference
Higher than normal	704/2628(26.79)		1.04(0.92–1.19)
Triglyceride level^b^		<0.0001	
Normal	2380/11072(21.50)		Reference
Higher than normal	1492/6188(24.11)		1.04(0.96–1.13)
LDL level^c^		<0.0001	
Normal	3117/14538(21.44)		Reference
Higher than normal	754/2721(27.71)		1.12(1.03–1.16)
HDL level^d^		0.9913	
Normal	3420/15246(22.43)		
Higher than normal	452/2014(22.44)		
BMI (kg/m^2^)		0.0028	
<18.5	168/908(18.50)		0.80(0.66–1.01)
18.5–24.0	1987/9046(21.97)		Reference
24.0–28.0	1254/5350(23.44)		1.07(0.98–1.17)
≥28.0	464/1960(23.67)		1.15(1.01–1.31)

Abbreviations: BMI = body mass index. BCG = Bacillus Calmette-Guerin. CI = confidence interval. HDL = high density lipoprotein. LDL = low density lipoprotein. OR = odds ratio. QFT = QuantiFERON-TB Gold In-Tube. TB = tuberculosis. T2DM = type 2 diabetes mellitus.

*Controlling for variables with p < 0.05 in the univariate analysis.

^a,b,c,d^Ranges of normal value for blood tests in different study sites: Cholesterol: Site A = 2.59–5.17 mmol/L, Site B = 3.1–5.17 mmol/L, Site C = 0–5.17 mmol/L, Site D = 0–5.17 mmol/L. Triglyceride: Site A = 0–1.7 mmol/L, Site B = 0.56–1.7 mmol/L, Site C = 0–1.71 mmol/L, Site D = 0–2.3 mmol/L. HDL: Site A = >0.9 mmol/L, Site B = 0.9–2.28 mmol/L, Site C = 1.16–1.55 mmol/L, Site D = 1.05–1.74 mmol/L. LDL: Site A = 0–4.11 mmol/L, Site B = 0–3.36 mmol/L, Site C = 0–3.12 mmol/L, Site D = <3.1 mmol/L.

**Table 3:** Potential independent factors associated with QFT positivity of the study population.

Variables	n/N(%)	p for χ^2^ test	Adjusted OR* (95% CI)
Study site		<0.0001	
Site A	1052/5014(20.98)		Reference
Site B	801/4141(19.34)		1.05(0.94–1.17)
Site C	1173/4213(27.84)		1.39(1.26–1.54)
Site D	847/3896(21.74)		1.31(1.17–1.46)
Gender		<0.0001	
Female	1793/9415(19.04)		Reference
Male	2080/7849(26.50)		1.30(1.17–1.44)
Age (years)		<0.0001	
18–29	194/2344(8.28)		Reference
30–39	313/2116(14.79)		1.84(1.51–2.25)
40–49	876/4513(19.41)		2.50(2.10–2.97)
50–59	934/3479(26.85)		3.85(3.24–4.59)
60–69	966/3047(31.70)		4.93(4.14–5.86)
≥70	590/1765(33.43)		5.60(4.65–6.74)
Smoking status		<0.0001	
Never smoked	2405/12176(19.75)		Reference
Ever smoked	1468/5086(28.86)		1.28(1.15–1.43)
History of close contact with TB patients		<0.0001	
No	3480/15826(21.99)		Reference
Yes	221/708(31.21)		1.38(1.16–1.65)
LDL level^a^		<0.0001	
Normal	3117/14538(21.44)		Reference
Higher than normal	754/2721(27.71)		1.14(1.02–1.27)
BMI (kg/m^2^)		0.0028	
<18.5	168/908(18.50)		0.89(0.76–1.03)
18.5–24.0	1987/9046(21.97)		Reference
24.0–28.0	1254/5350(23.44)		1.09(0.99–1.18)
≥28.0	464/1960(23.67)		1.17(1.04–1.33)

Abbreviations: BMI = body mass index. CI = confidence interval. LDL = low density lipoprotein. OR = odds ratio. QFT = QuantiFERON-TB Gold In-Tube. TB = tuberculosis.

^*^Controlling for study site, age, gender, smoking status, LDL level, history of close contact with TB, and BMI when appropriated.

^a^Range of normal value in different study sites: Site A = 0–4.11 mmol/L, Site B = 0–3.36 mmol/L, Site C = 0–3.12 mmol/L, Site D = <3.1 mmol/L.

**Table 4:** Association between QFT positivity and BMI (kg/m^2^) categories by sites.

BMI categories	QFT positivity		Adjusted OR† (95% CI)
	n/N	%	
Site A			
<18.5 kg/m^2^	41/217	18.89	0.98(0.68–1.41)
18.5–24.0 kg/m^2^	553/2755	20.07	Reference
24.0–28.0 kg/m^2^	363/1609	22.56	1.10(0.95–1.29)
≥28.0 kg/m^2^	95/433	21.94	1.04(0.81–1.34)
Site B			
<18.5 kg/m^2^	20/126	15.87	0.75(0.45–1.26)
18.5–24.0 kg/m^2^	350/1804	19.40	Reference
24.0–28.0 kg/m^2^	280/1384	20.23	1.00(0.83–1.20)
≥28.0 kg/m^2^	151/827	18.26	0.93(0.74–1.16)
Site C			
<18.5 kg/m^2^	58/279	20.79	0.67(0.48–0.92)
18.5–24.0 kg/m^2^	616/2281	27.01	Reference
24.0–28.0 kg/m^2^	360/1236	29.13	1.20(1.01–1.41)
≥28.0 kg/m^2^	139/417	33.33	1.52(1.20–1.94)
Site D			
<18.5 kg/m^2^	49/286	17.13	0.87(0.59–1.29)
18.5–24.0 kg/m^2^	468/2206	21.21	Reference
24.0–28.0 kg/m^2^	251/1121	22.39	1.00(0.82–1.20)
≥28.0 kg/m^2^	79/283	27.92	1.42(1.05–1.91)

Abbreviations: BMI = body mass index. CI = confidence interval. OR = odds ratio. QFT = QuantiFERON-TB Gold In-Tube. TB = tuberculosis.

^†^Controlling for age, gender, smoking status, LDL level, and history of close contact with TB patients.

**Table 5:** The association of BMI with QFT positivity stratified by various independent factors.

Variables	BMI	n/N(%)	Adjusted OR* (95% CI)
Gender			
Female	<28.0 kg/m^2^	1540/8643(17.82)	Reference
	≥28.0 kg/m^2^	253/1149(22.02)	1.26(1.08, 1.48)
Male	<28.0 kg/m^2^	1869/7142(26.17)	Reference
	≥28.0 kg/m^2^	211/861(24.51)	0.97(0.82, 1.15)
Age (years)			
<60	<28.0 kg/m^2^	2023/11308(17.89)	Reference
	≥28.0 kg/m^2^	294/1529(19.23)	1.03(0.90, 1.19)
≥60	<28.0 kg/m^2^	1386/4477(30.96)	Reference
	≥28.0 kg/m^2^	170/481(35.34)	1.31(1.07, 1.60)
Smoking status			
Never smoked	<28.0 kg/m^2^	2088/11140(18.74)	Reference
	≥28.0 kg/m^2^	317/1464(21.65)	1.18(1.03, 1.35)
Ever smoked	<28.0 kg/m^2^	1321/4643(28.45)	Reference
	≥28.0 kg/m^2^	147/546(26.92)	0.99(0.80, 1.22)
History of close contact with TB patients			
No	<28.0 kg/m^2^	3058/14414(21.22)	Reference
	≥28.0 kg/m^2^	422/1878(22.47)	1.11(0.99, 1.25)
Yes	<28.0 kg/m^2^	197/666(29.58)	Reference
	≥28.0 kg/m^2^	24/75(32.00)	1.17(0.696, 1.98)
LDL level^a^			
Normal	<28.0 kg/m^2^	2778/13396(20.74)	Reference
	≥28.0 kg/m^2^	339/1545(21.94)	1.11(0.97, 1.27)
Higher than normal	<28.0 kg/m^2^	631/2387(26.43)	Reference
	≥28.0 kg/m^2^	123/462(26.62)	1.12(0.88, 1.41)

Abbreviations: BMI = body mass index. CI = confidence interval. LDL = low density lipoprotein. OR = odds ratio. QFT = QuantiFERON-TB Gold In-Tube. TB = tuberculosis.

^*^Controlling for study site, age, gender, smoking status, LDL level, and history of close contact with TB.

^a^Range of normal value in different study sites: Site A = 0–4.11 mmol/L, Site B = 0–3.36 mmol/L, Site C = 0–3.12 mmol/L, Site D = <3.1 mmol/L.
