# Supplementary material for: Association of Body Mass Index with the Tuberculosis Infection: a Population-based Study among 17796 Adults in Rural China
Source: Sci Rep. 2017 Feb 8;7:41933. doi: 10.1038/srep41933 (PMC5296872; doi:10.1038/srep41933)
Supplement: Supplementary Table S1–Table S3 [file srep41933-s1.pdf]

Association of Body Mass Index with the Tuberculosis Infection: a Population-based Study among 17796 Adults in Rural China

Haoran Zhang[1], Xiangwei Li[1], Henan Xin[1], Hengjing Li[1], Mufei Li[1], Wei Lu[2], Liqiong Bai[3], Xinhua Wang[4], Jianmin Liu[5], Qi Jin[1]* & Lei Gao[1]*

[1]MOH Key Laboratory of Systems Biology of Pathogens, Institute of Pathogen Biology, and Centre for Tuberculosis, Chinese Academy of Medical Sciences and Peking Union Medical College, Beijing, China, 100730

[2] Jiangsu Provincial Center for Diseases Control and Prevention, Nanjing, China, 210009

[3] Hunan Provincial Institute of Tuberculosis Prevention and Control, Changsha, China, 410006

[4] Gansu Provincial Center for Diseases Control and Prevention, Lanzhou, China, 730000

[5] The Sixth People's Hospital of Zhengzhou, Zhengzhou, China, 450061

***Correspondence:** Prof. Lei Gao and Prof. Qi Jin, MOH Key Laboratory of Systems Biology of Pathogens, Institute of Pathogen Biology, and Centre for Tuberculosis, Chinese Academy of Medical Sciences and Peking Union Medical College, Beijing, China, 100730. Email: gaolei@ipbcams.ac.cn (GL), jinqi@ipbcams.ac.cn (JQ). Telephone: 8610-67828550, fax numbers: 8610-67828550.

Table S1. Distribution of BMI (kg/m^2) by age (years) and gender

		Total		18-29 years		30-39 years		40-49 years		50-59 years		60-69 years		≥70 years		p-value
		N	%	n	%	n	%	n	%	n	%	n	%	n	%	
Total																
	<18.5	927	5.21	280	11.71	90	4.12	118	2.54	101	2.80	158	5.04	180	9.88	<0.0001
	18.5-24.0	9362	52.61	1483	62.02	1182	54.17	2217	47.67	1762	48.77	1660	52.93	1058	58.07	
	24.0-28.0	5496	30.89	417	17.44	648	29.70	1694	36.42	1316	36.42	990	31.57	431	23.66	
	≥28.0	2010	11.30	211	8.82	262	12.01	622	13.37	434	12.01	328	10.46	153	8.40	
Male																
	<18.5	416	5.20	127	11.60	27	2.93	45	2.28	50	3.03	75	4.92	92	10.97	<0.0001
	18.5-24.0	4256	53.18	599	54.70	432	46.80	942	47.77	885	53.67	878	57.57	520	61.98	
	24.0-28.0	2470	30.86	232	21.19	321	34.78	727	36.87	558	33.84	456	29.90	176	20.98	
	≥28.0	861	10.76	137	12.51	143	15.49	258	13.08	156	9.46	116	7.61	51	6.08	
Female																
	<18.5	511	5.22	153	11.81	63	5.00	73	2.72	51	2.60	83	5.15	88	8.95	<0.0001
	18.5-24.0	5106	52.14	884	68.21	750	59.57	1275	47.59	877	44.65	782	48.54	538	54.73	
	24.0-28.0	3026	30.90	185	14.27	327	25.97	967	36.10	758	38.59	534	33.15	255	25.94	
	≥28.0	1149	11.73	74	5.71	119	9.45	364	13.59	278	14.15	212	13.16	102	10.38	

Abbreviation: BMI=body mass index.

Table S2. Distribution of BMI (kg/m^2) by study site and gender

	Site A n (%)	Site B n (%)	Site C n (%)	Site D n (%)
Total				
N*	5014	4141	4212	3896
<18.5	217(4.33)	126(3.04)	278(6.60)	286(7.34)
18.5-24.0	2755(54.95)	1804(43.56)	2281(54.15)	2206(56.62)
24.0-28.0	1609(32.09)	1384(33.42)	1236(29.34)	1121(28.77)
≥28.0	433(8.64)	827(19.97)	417(9.90)	283(7.26)
Male				
N	2303	2069	1933	1544
<18.5	86(3.73)	57(2.75)	134 (6.93)	132(8.55)
18.5-24.0	1265(54.93)	944(45.63)	1088(56.29)	862(55.83)
24.0-28.0	765(33.22)	691(33.40)	539(27.88)	439(28.43)
≥28.0	187(8.12)	377(18.22)	172(8.90)	111(7.19)
Female				
N	2711	2072	2279	2352
<18.5	131(4.83)	69(3.33)	144 (6.32)	154(6.55)
18.5-24.0	1490(54.96)	860(41.51)	1193 (52.35)	1344(57.14)
24.0-28.0	844(31.13)	693(33.45)	697(30.58)	682(29.00)
≥28.0	246(9.07)	450(21.72)	245(10.75)	172(7.31)

Abbreviations: BMI=body mass index.

*Sum might not always be in total because of missing data. Frequency of missing data did not differ significantly between sites.

Table S3. Distribution of T2DM by BMI (kg/m^2) categories

T2DM	Total	<18.5 kg/m^2	18.5-24.0 kg/m^2	24.0-28.0 kg/m^2	≥28.0kg/m^2	p for χ^2 test
No	16396(94.97)	887(5.41)	8745(53.34)	5005(30.53)	1759(10.73)	<0.0001
Yes	868(5.03)	21(2.42)	301(34.68)	345(39.75)	201(23.16)	

Abbreviations: BMI=body mass index. T2DM=type 2 diabetes mellitus.

Data are n (%).
